# Ultrastructure of Terpene and Polyphenol Synthesis in the Bark of *Cupressus sempervirens* After *Seiridium cardinale* Infection

**DOI:** 10.3389/fmicb.2022.886331

**Published:** 2022-05-27

**Authors:** Gianni Della Rocca, Alessio Papini, Isabella Posarelli, Sara Barberini, Corrado Tani, Roberto Danti, Salvatore Moricca

**Affiliations:** ^1^Istituto per la Protezione Sostenibile delle Piante, Consiglio Nazionale delle Ricerche (IPSP-CNR), Sesto Fiorentino, Italy; ^2^Dipartimento di Biologia (BIO), Università di Firenze, Firenze, Italy; ^3^Dipartimento di Scienze e Tecnologie Agrarie, Alimentari, Ambientali e Forestali (DAGRI), Università di Firenze, Firenze, Italy

**Keywords:** cypress, canker, phloem, infectious disease, TEM, resin, terpenoids

## Abstract

Cypress Canker Disease (CCD) pandemic caused by *Seiridium cardinale* is the major constraint of many *Cupressaceae* worldwide. One of the main symptoms of the disease is the flow of resin from the cankered barks. While inducible phloem axial resin duct-like structures (PARDs) have recently been characterized from an anatomical point of view, their actual resin production is still being debated and has never been demonstrated. Although the involvement of polyphenolic parenchyma cells (PP cells) in the bark of *Cupressus sempervirens* after *S. cardinale* infection was revealed in one of our previous studies using light microscopy, their evolution from the phloem parenchyma cells is yet to be clarified. This study investigated functional and ultrastructural aspects of both PARD-like structures and PP cells by means of more in-depth light (LM) and fluorescence microscopy (FM) combined with histochemical staining (using Sudan red, Fluorol Yellow, NADI Aniline blue black, and Toluidine blue staining), in addition to Transmission Electron Microscope (TEM). Two-year-old stem sections of a *C. sempervirens* canker-resistant clone (var. “Bolgheri”), artificially inoculated with *S. cardinale*, were sampled 5, 7, 14, 21, and 45 days after inoculation, for time-course observations. FM observation using Fluorol yellow dye clearly showed the presence of lipid material in PARD-like structures lining cells of the cavity and during their secretion into the duct space/cavity. The same tissues were also positive for NADI staining, revealing the presence of terpenoids. The cytoplasm of the ducts' lining cells was also positive for Sudan red. TEM observation highlighted the involvement of plastids and endoplasmic reticulum in the production of terpenoids and the consequent secretion of terpenoids directly through the plasma membrane, without exhibiting vesicle formation. The presence of a high number of mitochondria around the area of terpenoid production suggests that this process is active and consumes ATP. The LM observations showed that PP cells originated from the phloem parenchyma cells (and possibly albuminous cells) through the accumulation of phenolic substances in the vacuole. Here, plastids were again involved in their production. Thus, the findings of this work suggest that the PARD-like structures can actually be considered PARDs or even bark traumatic resin ducts (BTRD).

## Introduction

Cypress Canker Disease (CCD), also known as cypress bark canker, is a pandemic caused by the invasive necrotrophic fungus *Seiridium cardinale* (Wagener) Sutton and Gibson (Order: Xylariales, Family: Amphisphaeriaceae) affecting many *Cupressaceae* (Danti et al., 2013a). A native of California (Wagener, 1928, 1939; Della Rocca et al., 2011, 2013), this fungal pathogen has spread all around the world mainly through the commerce of infected plants (Della Rocca et al., 2019); this has subsequently caused severe loss and significant limitations to the cultivation of cypresses (genus *Hesperocyparis* and *Cupressus*), especially *Cupressus sempervirens* L. (Graniti, 1998; Danti and Della Rocca, 2017).

A genetic improvement program of *C. sempervirens* for resistance to CCD has been carried out in Europe since the 1970s (Raddi and Panconesi, 1981; Danti et al., 2011), due to this species' valuable role in the landscape and its multifunctionality, especially in Mediterranean countries (Xenopoulos et al., 1990; Della Rocca et al., 2007; Farahmand, 2020). As a result, a series of canker-resistant varieties of *C. sempervirens* have been patented (Panconesi and Raddi, 1990; Danti et al., 2006, 2013b).

In the breeding program of cypress genotypes, it was essential to study the metabolic, molecular, and anatomical mechanisms which underlie the resistance, or susceptibility, to the fungal disease. While the ultrastructural modifications in the phloem, cambium, phloem parenchyma, and ray parenchyma colonized by *S. cardinale* were described by Mutto and Panconesi, 1987, it has been observed that resistance of the common cypress to CCD relies on the tree's ability to produce a barrier that compartmentalizes the bark tissues invaded by the pathogen by building up a ligno-suberized boundary zone (LSZ), which is then followed by the formation of a necrophylactic periderm (NP) (Ponchet and Andreoli, 1990; Spanos et al., 1999). This is the result of the activation of metabolic pathways leading to the synthesis of suberin in bark tissues (Danti et al., 2018). Another host defense reaction against the pathogen (easily visible to the naked eye) is the more or less abundant emission of resin from the cankered bark (Danti et al., 2013a). This was found to be due to increased production of terpenoids and a qualitative change in their mixture in the bark tissues around the *S. cardinale* infection point (Achotegui-Castells et al., 2015, 2016).

Recently, Della Rocca et al. (2021) focused on infection-associated anatomical changes in the phloem of *C. sempervirens*. Specifically, the dynamic accumulation of polyphenolic parenchyma cells (PP cells) and the time course of phloem axial resin duct (PARD)-like structures that form during the initial phase of the infection were documented at the histological level using light microscopy. Notably, a faster and more intense reaction, from a quantitative point of view, occurred a few days after inoculation in a CCD-resistant cypress genotype compared to the susceptible one, in terms of the number of PP cells and PARD-like structures. Danti et al. (2018) showed that the host responses in terms of expression of suberin biosynthesis-related genes differ between bark wounding and bark wounding coupled with pathogen inoculation.

This work intends to increase the understanding of anatomical and cytological changes in the reaction in the bark tissues of common cypress induced by infection with the pathogen *S. cardinale*. Our study investigated the differentiation and genesis of PP cells and PARD-like structures through observations using Light (LM), Fluorescence (FM), and Transmission Electron Microscopes (TEM). Particular attention was paid to the synthesis of polyphenols in the PP cells and of resin in the PARD-like structures. LM investigations employed different dyes to clarify whether PARD-like structures actually synthesize terpenoids inside them and consequently can be considered as induced resin ducts.

## Materials and Methods

### Plants and Stem Inoculation Procedures

The experimental setup was similar to that of Della Rocca et al. (2021). A canker-resistant clone of *C. sempervirens* (PM322, patented with the name “Bolgheri”) from the IPSP-CNR clonal collection was used in this study. Forty-five 2-year-old ramets were grafted onto individual 1-year-old *C. sempervirens* seed rootstocks, grown in 4-liter pots containing a mixture of peat, compost, and perlite (3:1:1, v/v/v) under a shading tunnel. The *S. cardinale* isolate used for the stem inoculation was the same standard ATCC 38654 isolate adopted in Della Rocca et al. (2021). This isolate of the pathogen was grown on 2% PDA (Potato Dextrose Agar) Petri dishes for 14 days in the dark at 25 ± 0.7°C. Cypress ramets were stem inoculated in April 2019 following the cork-borer procedure described in Danti et al. (2014). At the beginning of the experiment, plants had a mean height of 80 ± 9 cm and a mean diameter of 1.4 ± 0.2 cm at the base of the stem. For LM, FM, and TEM observations, 30 out of 45 cypress plants were stem inoculated and 6 were only stem injured with the same cork borer (but without inserting the pathogen into the wounds), while 9 others were left intact (as controls).

### Sampling and Sample Preparation

Transversal sections, 3–4 mm thick, were sampled from the stems of the 45 ramets of *C. sempervirens* (30 inoculated, 6 wounded + 9 intact) to observe anatomical and cytological changes occurring in the bark tissues (composed of cambium, phloem, and phellogen-derived tissues) after fungal infection or wounding. For TEM observations, sections were sampled at 2 mm (above and below) from the inoculation point (IP), 5 days after being inoculated, and 2 mm (above or below) from the developing external bark necrosis, 21 days after inoculation. For LM and FM viewing, sections were sampled 2 mm above or below the developing bark necrosis, 45 days after the inoculation. Stem sections were also sampled from the intact ramets (the controls) on those same days. The transversal sections used for LM and FM analysis were divided into four to five sectors, whose external part was then soaked in resin (see Section Light Microscope and Fluorescence Microscope Observations). Instead, the TEM-intended sections were processed following the indications in Section Transmission Electron Microscope Observations. Furthermore, from the wounded or inoculated ramets, fresh stem sections were sampled 5 cm from the wound or the IP (above and below), 7 and 14 days after the artificial inoculation or wounding (see Section LM Observations of Fresh Sections for Anatomy Investigation and PARD Occurrence at a Distance From the IP or Wound) to verify the presence of PARD-like structures at a distance from the IP. From the inoculated ramets, 14 days after the inoculation, stem sections were also cut at the IP to verify the presence of the fungal hyphae in the bark tissues.

#### Light Microscope and Fluorescence Microscope Observations

For LM observations (45 days after inoculation), we used a Leitz DM-RB Fluo Light Microscope equipped with a digital Nikon DS-L1 camera. Cross-sections (one per ramet; six inoculated ramets + three intact) were fixed in FAA (formalin 4%, acetic acid 2%, and alcohol 70%) for 7 days at 4°C. The slides with the sections were washed in 70% alcohol for 2 h and dehydrated in an ethanol series of progressive concentrations (80, 95, and 100% ethanol) twice in each step. Pre-inclusions were done by mixing 100% ethanol and Technovit 7100 resin (Kulzer, Germany) 1:1, and then at a 1:2 ratio for 2 h in each step, at room temperature. Finally, the samples were soaked in a container with pure resin overnight. After 24 h of hardening, the samples were inserted inside sealed polypropylene capsules and embedded in a pure Technovit resin and hardener/catalyst (15:1 ratio) at room temperature. Slides of the resin including the stem were cut with a Heidelberg rotary ultramicrotome (Reichert-Jung OM U3) to produce semi-thin sections (4–6 μm).

These sections were then stained. Fluorol Yellow 088 was used to discern the presence of lipid material (Bundrett et al., 1991, as modified in Giuliani et al., 2020) within the lining cells of PARD-like structures to see whether lipid material had been secreted into the duct space/cavity. Sudan Red 7B (Lison, 1960) confirmed the presence of the fatty acid component of the resin. NADI staining was used to identify the presence of terpenoids (David and Carde, 1964); for this, we used fresh material and sections were produced with a cryostat at −20°C. Finally, we applied Toluidine blue black as a generic stain. However, the latter staining can be used at controlled pH to discriminate between phenols, lignin, cell walls, and resins thanks to metachromasy as reported by Ribeiro and Leitão (2020).

#### Transmission Electron Microscope Observations

For TEM observations (5 and 21 days after inoculations), we largely followed the method already described in detail in Mosti et al. (2013) and Papini et al. (2014). Stem sections (one per ramet; six inoculated ramets per sampling date + 3 intact as controls for each sampling date) of about 3 mm were fixed in 2.5% glutaraldehyde in 0.1 M phosphate buffer at pH 7.2 for 10 h (overnight) at 4°C. The samples were then post-fixed in 1% OsO4 in the same buffer for 1 h. Washing steps were executed after both the glutaraldehyde and the OsO4 steps. The samples were dehydrated in an ethanol series and then in propylene oxide, which was used to discriminate Spurr resin (Spurr, 1969) until the samples were embedded in pure resin. Subsequently, the samples were sectioned with an ultramicrotome (Reichert-Jung ULTRACUT E). Sections of about 70 nm were stained with uranyl acetate and lead citrate. These sections were then examined with a Philips 201 TEM at 80 kV. TEM was used to assess ultrastructural changes in the phloem's albuminous cells upon their transformation into PP cells and during the PARD-like structures' development. Particular attention was given to plastid and vacuole changes, the accumulation of polyphenols, and terpene production to fill the PARD-like structures.

#### LM Observations of Fresh Sections for Anatomy Investigation and PARD Occurrence at a Distance From the IP or Wound

Fresh transversal sections of stems (one per ramet; six inoculated ramets per two sampling dates + 3 wounded ramets per date) cut 5 cm above and 5 cm below the IP or the wound were also observed to verify the formation of PARD-like structures at a distance from the lesion (inoculation or wounding). Intact control ramets were not used in this assay, as the recent work by Della Rocca et al. (2021) evidenced the absence of constitutive PARD-like structures in the bark of *C. sempervirens* clones. Stem segments were sampled 7 and 14 days after the treatment (inoculation or wounding) to verify the occurrence of a signal at a distance from the point of inoculation or wound. Semi-thin sections (1–5 μm) were stained with aniline blue black. Samples were observed and digitally imaged with a Leica DMRB Fluo microscope. Three portions of each section were observed at a magnification of 100 × for each sampling date (7 and 14 days) and for each of the three sampling sites (IP or wound, 5 cm above the IP or wound, and 5 cm below the IP or wound). For each portion, three pictures were taken to include the cambium (placed parallel to the X-axis) and the number of PARD-like structures (regardless of their developmental phase) was counted.

### Statistics

Differences in the number of PARD-like structures counted in the fresh stem sections of *C. sempervirens* ramets at the IP or wound and at 5 cm distance (above and below) after 7 and 14 days were analyzed by one-way ANOVA to evaluate the host's reaction at a distance from the stimulus at different times. Differences among means were compared using Tukey's honestly significant difference (HDS) *post-hoc* test for *p* < 0.05 using STATISTICA 10.0 software.

## Results

### Anatomy at IP and PARD-Like Structure Formation at a Distance From IP (LM)

**Figure 1:**
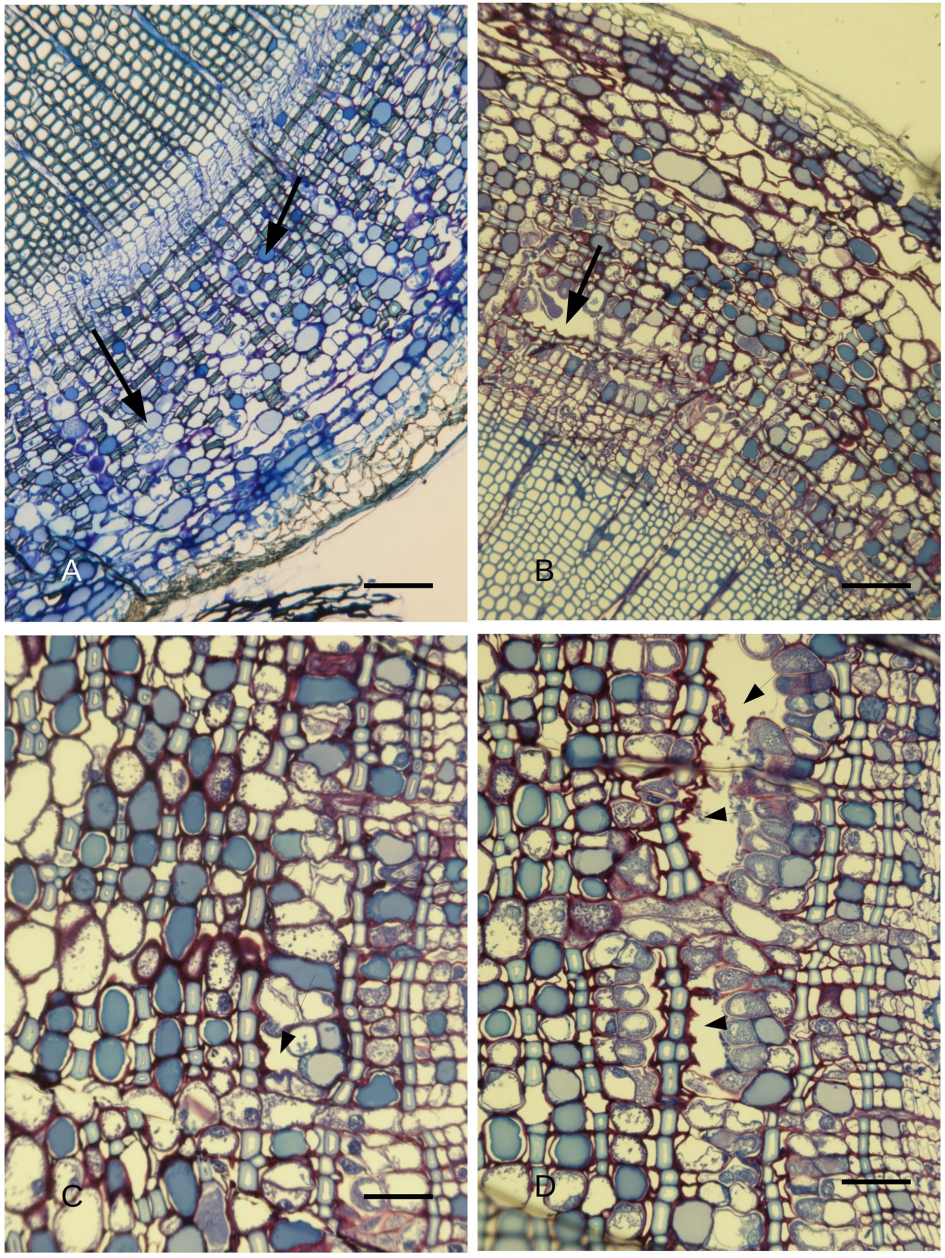
**(A)** *C. sempervirens* secondary cross-section of an intact stem (control) observed *via* light microscope after toluidine blue staining. No PARD-like structures can be observed in the phloem, while PP cells (two of them indicated with arrows) are numerous. Bar = 50 μm. **(B)** *C. sempervirens* secondary stem cross-section sampled 14 days after the infection with *Seiridium cardinale* observed at LM after toluidine blue staining. A PARD-like structure is formed in the secondary phloem belt. Bar = 50 μm. **(C)** *C. sempervirens* secondary stem cross-section, cambium zone. Toluidine blue staining. A phloem element degenerated leaving a small cavity (arrow) between a sclerenchymatic fiber and an albuminous cell (asterisk). PP cells are stained in dark blue, while sclerenchymatic fiber walls are light blue. Bar = 50 μm. **(D)** *C. sempervirens* secondary stem cross-section, cambium zone, 12 days after image in **(C)**. Toluidine blue staining. The cavities (arrows) are increasing in dimension due to the degeneration of phloem elements leading to the formation of a PARD. The albuminous cells along the side of the PARD increase their toluidine blue positivity. Bar = 50 μm.

At low magnification, a comparison between a secondary intact stem section (Figure 1A) and a stem section sampled from an inoculated stem 14 days after infection (Figure 1B) showed that the latter had formed a PARD-like structure, while the former was lacking these structures in the phloem. After 7 days from the artificial inoculation with the pathogen, well-developed PARD-like structures were equally observed in stem sections at the IP as well as at 5 cm above and below the IP, with no significant differences in their number between the three sampling points (4.8, 5, and 4.3 PARD-like structures averaged for each picture, respectively). Similarly, PARD-like structures were also observed in wounded ramets, but they were significantly fewer (*p* < 0.05) than in the inoculated ones after 7 days (3.7, 3.3, and 3.5 PARD-like structures on average for each picture at IP, 5 cm over, and 5 cm below the IP, respectively). At 14 days, the number of PARD-like structures was the same as after 5 days in both inoculated or wounded ramets. The formation of PARD-like structures occurred starting with the degeneration of phloem elements between the two rows of sclerenchymatic fibers (Figure 1C). Since, between two rows of sclerenchymatic fibers, there are two rows of phloem elements separated by an albuminous cell (**Figure 3**), the degeneration of the phloem element leaves a small cavity. This later grows as the nearby phloem elements continue to disintegrate (Figure 1D). Moreover, the albuminous cells adjacent to the PARD-like structure increased their toluidine blue positivity.

### PARD-Like Structures: Evidence of Resin Secretion (LM and FM)

**Figure 2:**
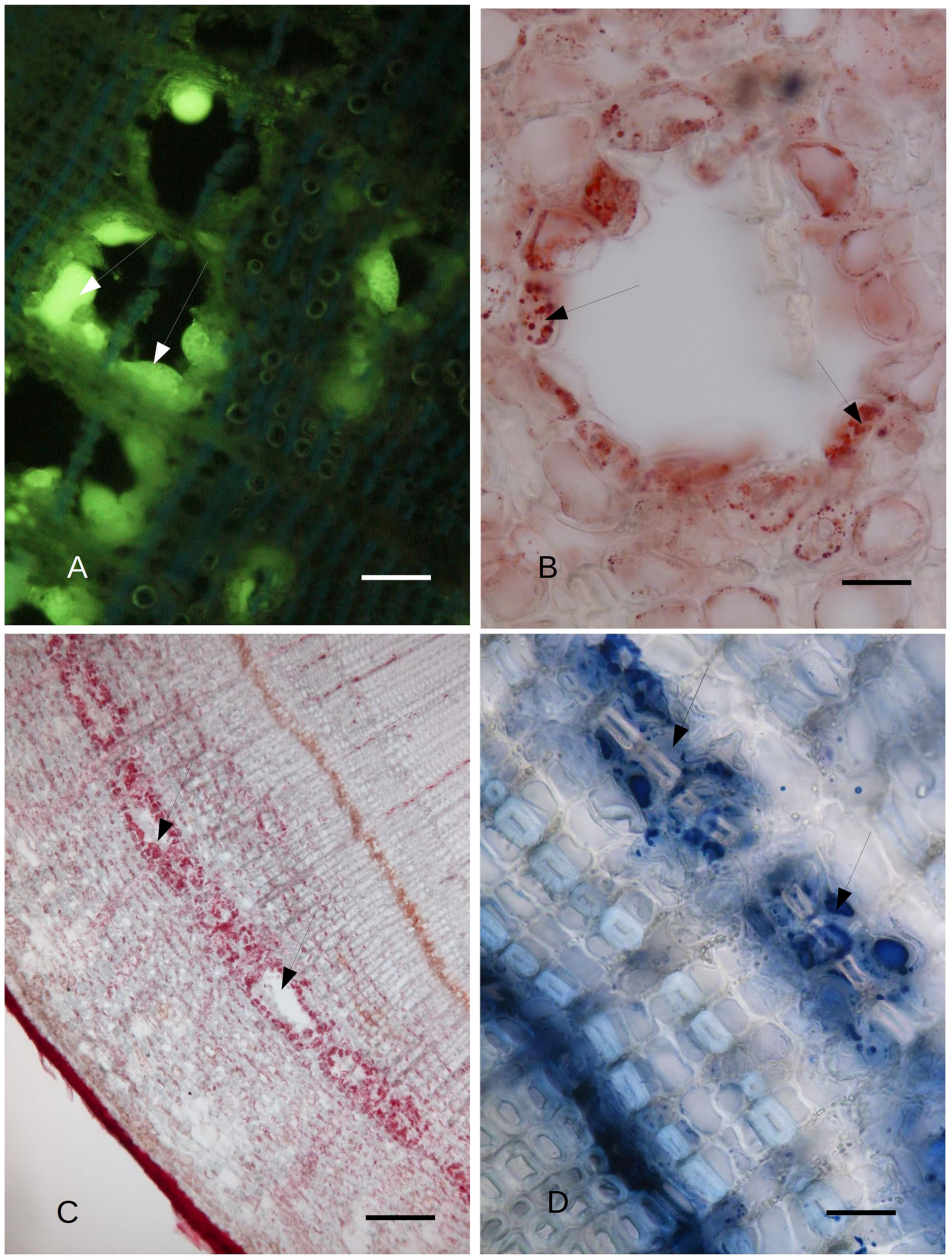
**(A)** *C. sempervirens* secondary stem cross-section 2 mm from the necrotic lesion caused by the *Seiridium cardinale* fungal pathogen. Bark tissues near the cambium zone were stained with Fluorol Yellow, 45 days after inoculation with the fungal pathogen, as observed by FM. The cells surrounding the cavities of the PARD-like structures react intensely to Fluorol Yellow (arrows), thus showing their lipophilic content. Bar = 40 μm. **(B)** Detail of the cross-section of a PARD-like structure stained with Sudan red. The cells lining a lacuna react with Sudan red, showing a lipophilic content in droplets. Bar = 20 μm. **(C)** Cavities (arrows) formed by the fusion of multiple PARD-like structures along the cambium in *C. sempervirens* 45 days after artificial inoculation with *S. cardinale*. The stem cross-section was cut 2 mm above the necrotic lesion caused by the fungal pathogen. The cells lining the cavities are strongly positive for Sudan red. Bar = 100 μm. **(D)** The highest NADI positivity was around the forming PARD-like structures (arrows) along the cambium in a stem cross-section of *C. sempervirens* 45 days after artificial inoculation with *S. cardinale*. The newly generated PARD-like structure is still subdivided into two cavities by a sclerenchymatic fiber. The stem section was cut 2 mm above the necrotic lesion induced by the pathogen. Bar = 40 μm.

**Figure 3:**
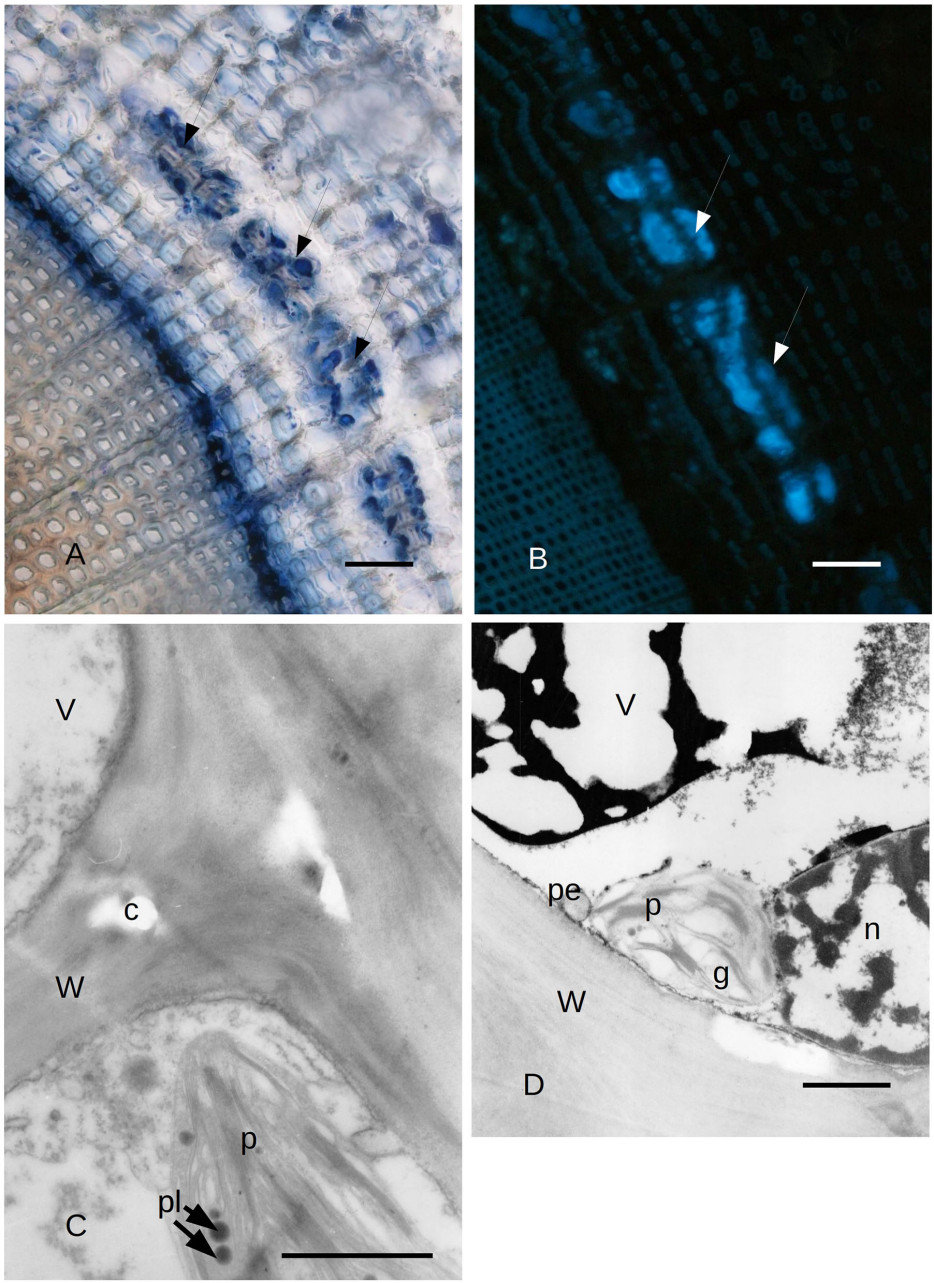
**(A)** Bark tissues near the cambium zone of *C. sempervirens* secondary stem cross-section, 2 mm from the necrotic lesion developed by the fungal pathogen *S. cardinale* 45 days after artificial inoculation. The NADI positive cells (arrows) are in the same position as those positive to Sudan red in Figure 5. Bar = 80 μm. **(B)** Autofluorescent material emitting blue UV light inside the same cells and cavities that were NADI positive in Figure 2. Bark tissues near the cambium zone of *C. sempervirens* (secondary stem cross-section) cut 2 mm from the necrotic lesion developed by the fungal pathogen *S. cardinale* 45 days after artificial inoculation. Bar = 100 μm. **(C)** TEM image of *C. sempervirens* bark tissues of control plants (intact). The albuminous cells in the secondary phloem show active plastids (p) and some plastoglobules (pl). Some crystals (c) are forming in the wall (W) (V, vacuole). Bar = 1 μm. **(D)** TEM image of cross-section of *C. sempervirens* bark tissues of intact plants (controls) (V, vacuole; g, starch granule; n, nucleus; W, cell wall). PP cell in the secondary phloem; a chloroplast (p) appears still active and near peroxisome (pe). Polyphenols are forming inside the vacuole. Bar = 1 μm.

The cypress stem sections cut just above and below the pathogen-induced bark necrosis at 45 days after inoculation were stained with Fluorol Yellow; these showed the presence of lipid/lipophilic material within large lacunae of the PARD-like structure in the secondary phloem layer (Figure 2A). Part of the Fluorol Yellow positivity was inside the cavity. This result was confirmed by Sudan red staining (Figures 2B,C). Sudan red positivity was due to the presence of small particles inside the cells surrounding the cavity (Figure 2B). Furthermore, a discontinuous circle of cavities formed around the lacunae outside the cambium (Figure 2C). NADI reagent showed that the lipidic material inside the cavities and the cells surrounding the cavity contained terpenoids (Figures 2D, 3A). In Figure 2B, the lacuna also contained NADI-positive material and was divided by one sclerenchymatic fiber. The NADI-positive cavities (Figure 3A) generally were in the same position as the Sudan red positive cavities seen in Figure 2C. The same NADI-positive material also exhibited autofluorescence with UV blue light (Figure 3B).

### Observations *via* Transmission Electron Microscope

**Figure 4:**
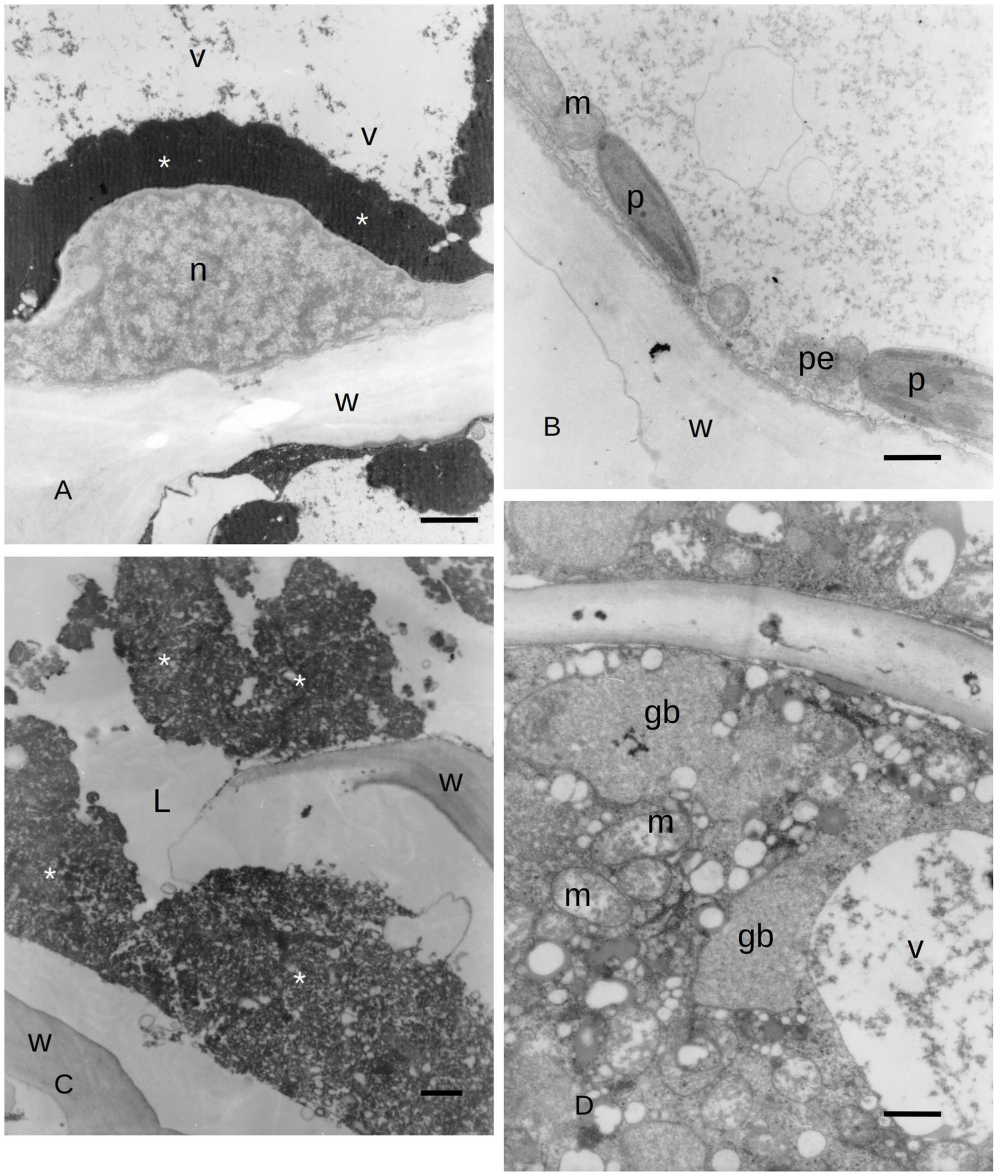
**(A)** TEM image of PP cells with polyphenols inside (asterisks) the secondary phloem of *C. sempervirens* 5 days after infection with the fungal pathogen *S. cardinale*. (V, vacuole; n, nucleus; W, cell wall) Bar = 1 μm. **(B)** TEM image of an albuminous cell with active plastids (p) near mitochondria (m) and peroxisomes (pe) in the secondary phloem of *C. sempervirens* 5 days after infection with the fungal pathogen *S. cardinale*. (W, cell wall) Bar = 1 μm. **(C)** TEM image of a PARD-like structure with the lumen (L) partially occupied by granular material (asterisks) and remnants of cell walls in bark tissues of *C. sempervirens* 21 days after inoculation with *S. cardinale* (W, cell wall) Bar = 1 μm. **(D)** TEM image of *C. sempervirens* bark tissues 21 days after infection with the pathogen *S. cardinale*. Detail of the epithelial cell of a PARD-like structure with active mitochondria (m) and vacuoles (v) filled with granular material (gb, gray body) Bar = 1 μm.

**Figure 5:**
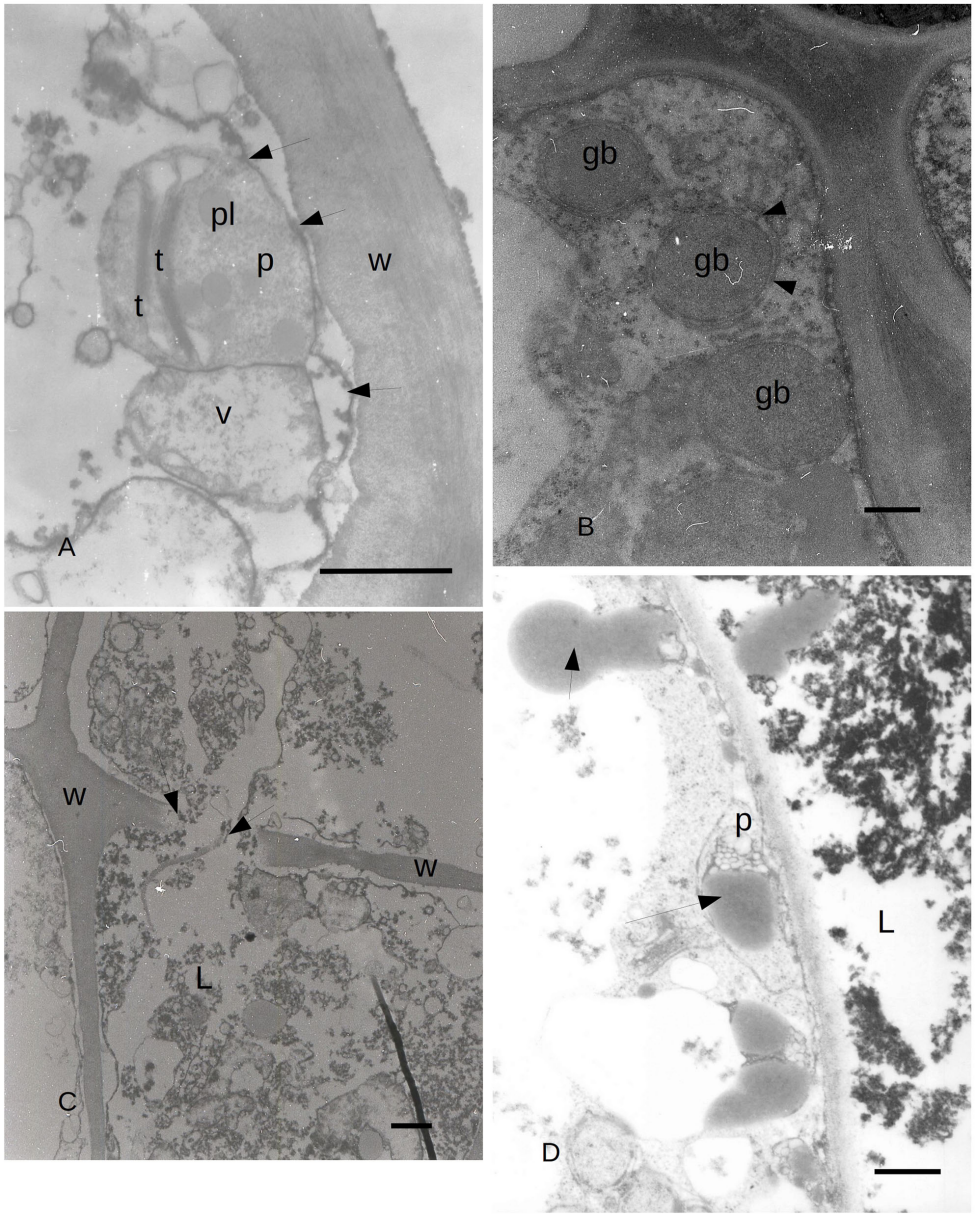
**(A)** TEM image of *C. sempervirens* bark tissues 21 days after infection with the pathogen *S. cardinale*. PARD-like structure epithelial cells. Plastid (p) degeneration with few thylakoids (t) still intact, with lipid bodies and granular material. Vacuoles (V) are in contact with the plastid, while ER elements (arrows) begin to surround them (W, cell wall; pl, plastoglobules) Bar = 1 μm. **(B)** TEM image of PARD-like structure in *C. sempervirens* bark tissues 21 days after infection with the pathogen *S. cardinale*. Detail of the epithelial cell with gray bodies (gb) surrounded by endoplasmic reticulum (arrows) and ribosomes as gray dots in the cytoplasm. Bar = 1 μm. **(C)** TEM image of *C. sempervirens* bark tissues 21 days after infection with the pathogen *S. cardinale*. Mature PARD-like structure and activity of the epithelial cell. PARD-like structures are merging, while the wall fractures (arrows) (W, cell wall; L, lumen) Bar = 1 μm. **(D)** TEM image of *C. sempervirens* bark tissues 21 days after infection with the pathogen *S. cardinale*. Production and extrusion of gray material (arrows) from the epithelial cell into the PARD-like structure lumen (L) (p, plastid) Bar = 500 nm. Gb, gray bodies; L, lumen; p, plastid; pl, plastoglobule; t, thylakoid; V, vacuole; W, cell wall.

Due to the results obtained with LM, we focused our attention on the ultrastructure of the albuminous cells and the surrounding cells at 5 and 21 days after inoculation (compared to the control and uninoculated plants). Before the infection with *S. cardinale*, the albuminous cells showed plastids with apparently functional thylakoid apparatus containing plastoglobules, whose crystals were forming in the cell wall (Figure 3C). The formation of PP cells began with the accumulation of very dense material along the tonoplast of the large vacuole (Figure 3D). The nucleus showed condensed chromatin and the plastids contained large granules of starch, while the thylakoid apparatus was arranged in small grana (Figure 3D), which were smaller and less organized than those in the albuminous cell seen in Figure 3C. Some peroxisomes were visible near the plastids (Figure 3D). Five days after the inoculation, the thence-formed PP cells showed vacuoles with abundant electron-dense material along the tonoplast and granular material inside the vacuolar space, while the nucleus seemed to be less active and electron dense (Figure 4A) compared to the albuminous and parenchyma cells. In the albuminous cells, for example, the plastids were at the periphery, touching the peroxisomes and mitochondria (Figure 4B). Twenty-one days after inoculation, the PARD-like structures (corresponding to the cavities observed with LM) appeared partially occupied by cellular remnants (in particular, cell walls) and granular material (Figure 4C). Epithelial cells (around the PARD-like structure) were almost completely occupied by small to medium gray bodies with granular content, apparently derived from small vacuoles, together with even smaller vacuoles with electron transparent content (Figure 4D). Also, a larger vacuole was present (Figure 4D). The most easily recognizable organelles were mitochondria, which showed rarefied cristae and were arranged around the gray bodies (Figure 4D). The plasma membrane appeared detached from the wall at some points (Figure 4D). At a later stage, in the epithelial cells, the plastids began to degenerate, losing almost completely their thylakoids, forming internal lipid bodies and granular material (Figure 5A). They entered into contact with vacuoles approximately the same size as the plastid, while endoplasmic reticulum (ER) elements began to surround them (Figure 5A). In other cells, apparently at a yet later stage of development and secretion, most organelles were no longer recognizable individually in the cytoplasm. Roundish gray bodies formed close to the cell wall and were surrounded by ER (Figure 5B). At an even later stage, the PARD-like structure began to fill with granular material, while the walls separating different cells forming the PARD-like structure began to collapse (Figure 5C). Gray material started forming within the organelles of the epithelial cells, but then was found free in the cytoplasm, and finally outside the epithelial cell in the lumen of the PARD-like structure (Figure 5D).

## Discussion

This work investigated the genesis and the evolution of PP cells and PARD-like structures in *Cupressus sempervirens* in the phloem tissues after inoculation with the fungal bark pathogen *S. cardinale*. Their developments were examined at intervals, from 7 to 45 days after infection with the pathogen, at both anatomical and cytological levels *via* LM, FM, and TEM using different dyes and techniques. In Della Rocca et al. (2021), the involvement of both PARD-like structures and PP cells in the response of *C. sempervirens* to the necrotrophic bark pathogen *S. cardinale* had been found in both canker-resistant and -susceptible clones. This study evidenced a faster phloem response of the canker-resistant clone in terms of the number of PP cells and PARD-like structures formed 5 and 12 days after infection compared to the susceptible clone.

Resin production is one of the main mechanisms of defense in conifers (Krokene et al., 2008a). Conifer oleoresin is a complex mixture of volatile mono- (C10) and sesquiterpenes (C15), as well as non-volatile diterpene and resin acids. It accumulates at the wound site to counteract invaders (insects and fungi) in several ways: through some antimicrobial activity, by flushing the wound, and by sealing the injury (Phillips and Croteau, 1999; Trapp and Croteau, 2001; Zulak and Bohlmann, 2010).

As reported in Krokene et al. (2008a), *Cupressaceae* do not have pre-formed resin structures, while induced structures appear following a stimulus to the xylem (*Sequoia, Sequoiadendron, Metasequoia*) or the phloem (*Cupressus, Hesperocyparis, Chamaecyparis, Cryptomeria*). *C. sempervirens* develops PARD-like structures as a reaction to the infection of the bark canker agent *S. cardinale* (Moriondo, 1972; Ponchet and Andreoli, 1990; Della Rocca et al., 2021); its main external symptom is more or less intense resinosis by the cankered bark (Danti et al., 2013a). Although the association between resinosis and infection by pathogenic fungi has been reported (Madar et al., 1995; Spanos et al., 1999; Achotegui-Castells et al., 2015, 2016; Danti et al., 2018), the recent work by Della Rocca et al. (2021) has not definitively clarified the resin production site, perhaps because of the short duration of the experiment. For this reason, one of the goals of this research was to investigate in depth the hypothesis that PARD-like structures are the true sites where resin is produced by the host, whether as a reaction to a fungal pathogen invading the bark tissues or to wounds. Our observation, at first, indicated a quick reaction by the host to the infection/wound signal, after only 7 days from the injury stimulus. The PARD-like structures were produced not only at the IP but also 5 cm above and below the inoculation/wound point, thereby showing a “signal speed” of almost 1 cm per day (at least in the first phase of the stimulus). This is the first case reported of phloematic resin structure being remotely induced in the first days after a “traumatic event” (either pathogen infection or wound) in *Cupressaceae*. The presence of resin ducts in *C. sempervirens* barks affected by *S. cardinale* was previously observed in various works (Moriondo, 1972; Ponchet and Andreoli, 1990; Spanos et al., 1999; Achotegui-Castells et al., 2015, 2016; Danti et al., 2018). Madar and Liphschitz (1989) also reported the formation of traumatic resin ducts in *C. sempervirens* bark upon challenge by *S. cardinale* and *Diplodia cupressi*. The formation of resin ducts in the bark after fungal inoculation was also reported in *C. obtusa* (Yamanaka, 1989; Suto, 1998; Kusumoto and Suzuki, 2003; Fujii et al., 2018) and in *C. macrocarpa, Cryptomeria japonica, Sequoiadendron giganteum*, after stem treatment with methyl jasmonate (Hudgins and Franceschi, 2004). However, none of these studies reported the formation of traumatic resin ducts in the bark at a distance from the inoculation or wounding site.

Regarding the rapidity of the formation of phloematic resin ducts upon a traumatic event, a timing similar to that observed on *C. sempervirens* in this work is that reported for *C. obtusa* by Yamanaha (1989) and by Kusumoto and Suzuki (2003). In these two studies, the presence of phloematic resin structures was observed 7–15 days after wounding. All the other studies do not allow a direct comparison because they were not based on time series observations but on the detection of phloem resin ducts after arbitrary intervals of time, after infection, wounding, or treatment with methyl jasmonate. In this study, a lower number of PARD-like structures was also observed in the wounded ramets compared to the inoculated ones. Similar results were obtained by Luchi et al. (2005) in *Pinus nigra*, although in that case, they were xylematic traumatic resin ducts (TRDs). In that study, the non-aggressive fungal canker agent, *Diplodia scrobiculata*, artificially inoculated into the stem, induced in 12 days a higher number of TRDs per surface unit compared to those caused by mock inoculations (with sterile agar). In addition, Luchi and fellow authors observed that TRD numbers declined as the sampling distance increased from the IP. One might however speculate that the anatomical modifications (i.e., the induction of resiniferous structures) at the phloematic level (as in *C. sempervirens*) might be a little faster than at the xylematic level (as in *Pinus nigra*). The remote induction of TRDs has been also observed in *Picea abies* by Franceschi et al. (2000), Nagy et al. (2000) and Krekling et al. (2004). Surprisingly, the serious canker agent *Sphaeropsis sapinea* was unable to induce TRD in the xylem of *P. nigra* (Luchi et al., 2005). This might indicate that the induction of TRDs may be a result of a transmittable signal that may relate to perception by the host of fungus or fungal activity.

Due to the degeneration of some phloem elements and ray parenchyma, in this study, we also observed, 45 days after inoculation, a sort of tangential coalescence of neighboring PARD-like structures, which thus formed larger cavities. Coalescence of neighboring PARD-like structures had already been described by Suto in *Chamaecyparis obtusa* 1 month after inoculation with the fungal pathogen *Cistella japonica* (2005). A similar process was found in *Thuja plicata* by Cleary and Holmes (2011), although with the difference that in *T. plicata*, elliptical zones of phellem-like cells in degeneration and necrosis were observed around the longitudinal phloematic resiniferous ducts, which does not occur in the phloem of *Cupressus*.

**Figure 6:**
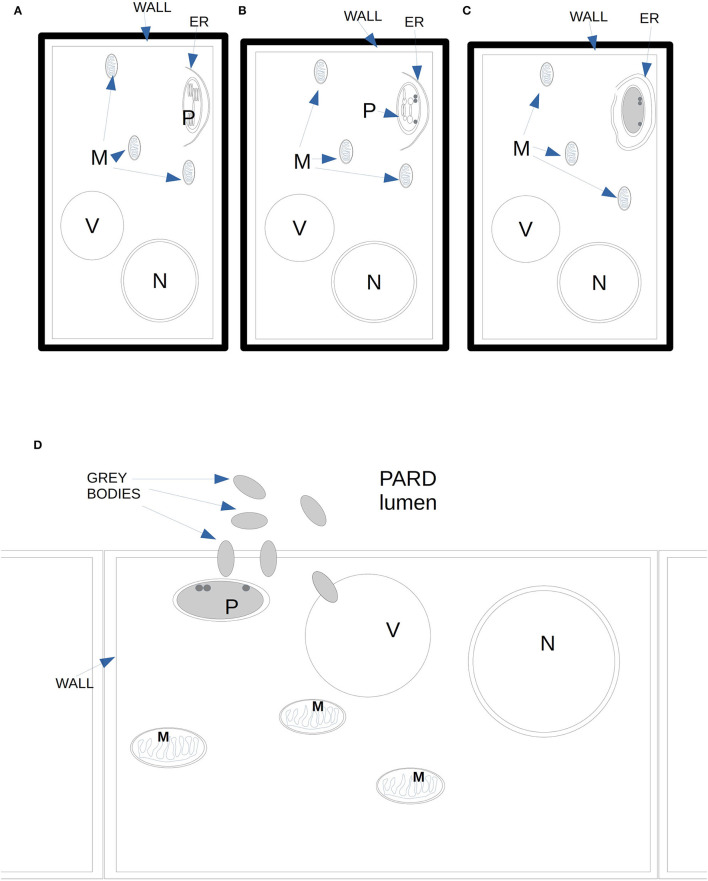
Diagram of the proposed four stages of gray bodies development in an epithelial cell bordering the PARD lumen. **(A)** Close to the plasma membrane on the side of the PARD lumen, a plastid with normal thylakoids starts to enter in contact with ER elements. **(B)** In the plastid, the thylakoids reduce in number and tend to dilate the margins of their cisternae. **(C)** The ER surrounds almost completely the plastid whose stroma is filled with granular content. Some darker globules can be observed on the side of the plasma membrane. No thylakoid is visible anymore. **(D)** Gray bodies start to cross the plastid membranes and even the plasma membrane, ending finally in the PARD lumen.

During the activity of PARD-like epithelial cells, different phases could be observed at the same moment in adjacent cells, which showed a different electron-dense cytoplasm. The increased electron density was probably due to a greater number of ribosomes involved in protein synthesis. Similarly, in *Picea abies*, the epithelial cells of xylem TRD exhibited a greater cytoplasmic density, a high number of plastids, and enlarged nuclei, as observed by Nagy et al. (2000). Apart from the vacuole, the organelle mostly involved in the synthesis of terpenes was the chloroplast which was almost surrounded by a smooth ER; therefore, the formation of terpenes was to be related to the interaction between chloroplast and endoplasmic reticulum. The produced terpenes tended to get out of the cytoplasm by passing through the plasmalemma apparently without the need for specific transport mechanisms. Terpenes in the form of gray bodies were also observed between the plasmalemma and epithelial cell wall. We propose here a possible model of terpenoid formation (Figure 6) starting from plastids, with four sequential stages (Figures 6A–D), based on the observation of gray bodies *via* TEM. The involvement of the ER in the modification of terpenes had already been proposed by Berthelot et al. (2012), even if without any evidence of an ER-plastid spatial colocalization, while Mehrshahi et al. (2013) showed the importance of ER in modifying the terpenoid tocopherol (with fatty acid desaturation) produced by chloroplasts. The constant presence of mitochondria even in the advanced stages of PARD-like epithelial cell maturation shows that the production of terpenes is an active mechanism that occurs with ATP consumption. Finally, in PARD-like structures, there are also cellular residues derived from the degeneration of fibers and phloem cells, that are being lysed to allow the enlargement of the PARD-like structure lumen *via* wall digestion. The involvement of mitochondria in the last phases of PCD, in general, and in plants, in particular, has been often observed as a consequence of an active PCD process (Adrain and Martin, 2001; Yao et al., 2004; Brighigna et al., 2006).

The content of the PARD-like structure in *C. sempervirens* was confirmed to be lipophilic (*via* Sudan red and Fluorol Yellow positivity) and more specifically composed of terpenoids, as showed by NADI staining positivity and the autofluorescence with UV leading to blue light emission, which may be due to diterpenes and/or oleoresins (Donaldson and Williams, 2018; Donaldson, 2020). About the nature of the terpenes contained in the PARD-like structure, Li et al. (2021) tried to link a specific intensity of NADI staining to a particular class of molecules belonging to terpenes. The topic has not yet been well explored, however, in *Cupressus*, the NADI staining intensity here observed in the PARD-like structures appeared to be more similar to that corresponding to terpenes similar to (E)-β-farnesene found in pyrethrum, even if the exact identification of the specific molecule is not possible with histochemistry.

The PP cells have been identified as another form of defense of *C. sempervirens* as well as a potential proxy of resistance to CCD (Della Rocca et al., 2021), demonstrating that in *C. sempervirens*, new PP cells can originate from albuminous cells through the accumulation of phenolic substances in the vacuole, as noted by Fahn (1990), Krekling et al. (2000), and Krokene et al. (2008b) in *Picea abies*. Their origin, however, had never been investigated before at an ultrastructural level. Although Della Rocca et al. (2021) observed a decrease of PP cells during the formation of the PARD-like structures, probably because some PP cells had lost their vacuolar polyphenol content, similarly to what was observed in *Picea abies* by Nagy et al. (2000). In our study, we observed a few albuminous cells starting a differentiation process 5 days after infection, and then forming what would then become the PARD-like epithelial cells. These cells were very active, with some rounded plastids that formed intra-plastid lipid bodies. Other albuminous cells showed polyphenol accumulations in the middle of the vacuole. TEM observations clarified the ultrastructure of the PP cells in *C. sempervirens*, better distinguishing their genesis. The albuminous cells exhibited low electron-dense cytoplasm; the plastids were located at the periphery of the cytosol, in contact with peroxisomes and mitochondria. In these cells, the plastids were particularly active, showing thylakoids arranged into grana, more plastoglobules (defined as in Van Wijk and Kessler, 2017), and with less accumulated starch compared to PP cells during formation. The constitutive PP cells showed a large vacuole that occupied almost the entire cell volume in which electron-dense material was identified as polyphenols. In the periphery of these cells, the cytoplasm was very dense and we recognized dictyosomes (whose function is to secrete vesicles, which generally contain carbohydrates or glycoproteins, often outside the cell) and plastids with plastoglobules. Plastids are involved in the production of polyphenol precursors, and the key enzyme of their biosynthesis has been localized on the chloroplast starch granules (Grundhöfer et al., 2001). TEM observations showed that the plastids observed in the PP cells of the common cypress contained large starch granules, similarly to what Krekling et al. (2000) observed in PP cells of *Picea abies*. The same authors also detected the presence of lipids, hypothesizing that both compounds constituted an energy reserve for a rapid synthesis of defense compounds (Krokene et al., 2008a). Within PP cells, some peroxisomes, probably involved in lipid metabolism, were also visible. The newly induced PP cells showed vacuoles with a higher electron density and granular material inside compared to the constitutive PP cells. In more advanced stages of maturation, instead, PP cells showed an increased amount of polyphenols in the vacuoles, suggesting that the host defense response against the infection could be related to the total quantity of polyphenols accumulated around the affected area.

## Conclusions

In *C. sempervirens*, both PP cells and resins producing cells in PARD structures can be induced as a response to an attack by a pathogen. Both these cell types develop out of albuminous or parenchyma cells in the secondary phloem layer of the stem's secondary structure.

Plastids appear to be involved in the development of the PP cells, with a phase of starch accumulation and a later stage in which the majority of the cells are filled by a vacuole almost completely occupied by polyphenols.

The resin-producing cells surrounding the PARDs secrete terpenoids toward a large lumen formed by programmed cell death of albuminous cells and parenchyma cells around sclerenchymatous fibers. Terpenoids are produced inside the cells after the ER surrounds the plastids, then the secretion occurs directly through the plasma membrane toward the lumen.

This study showed the production of terpenoids in the lining cells surrounding the lumens which formed in the phloem of *C. sempevirens* following both infections with *S. cardinale* or mechanical injury. These findings suggest that the structures previously defined as PARD-like can be considered traumatic resin ducts of the bark (BTRD).

## Data Availability Statement

The raw data supporting the conclusions of this article will be made available by the authors, without undue reservation.

## Author Contributions

GD, AP, and RD conceived the idea and designed the methodology. CT and AP performed the microscope observations. SB and IP carried out the inoculations, sampling, and preparation of samples for microscope observations. GD, AP, RD, SB, and SM analyzed the data and wrote the manuscript. All authors contributed critically to the drafts and gave final approval for publication.

## Conflict of Interest

The authors declare that the research was conducted in the absence of any commercial or financial relationships that could be construed as a potential conflict of interest.

## Publisher's Note

All claims expressed in this article are solely those of the authors and do not necessarily represent those of their affiliated organizations, or those of the publisher, the editors and the reviewers. Any product that may be evaluated in this article, or claim that may be made by its manufacturer, is not guaranteed or endorsed by the publisher.

## References

[B1] Achotegui-Castells A. Danti R. Llusià J. Della Rocca G. Barberini S. Peñuelas J. (2015). Strong induction of minor terpenes in Italian Cypress, *Cupressus sempervirens*, in response to infection by the fungus *Seiridium cardinale*. J. Chem. Ecol. 41, 224–243. 10.1007/s10886-015-0554-1 25740205

[B2] Achotegui-Castells A. Della Rocca G. Llusià J. Danti R. Barberini S. Bouneb M.. (2016). Terpene arms race in the *Seiridium cardinale*–*Cupressus sempervirens* pathosystem. Sci. Rep. 6, 18954. 10.1038/srep18954 PMC4726198 26796122

[B3] Adrain C. Martin S. J. (2001). The mitochondrial apoptosome: a killer unleashed by the cytochrome seas. Trends Biochem. Sci. 26, 390–397. 10.1016/S0968-0004(01)01844-8 11406413

[B4] Berthelot K. Estevez Y. Deffieux A. Peruch F. (2012). Isopentenyl diphosphate isomerase: a checkpoint to isoprenoid biosynthesis. Biochimie. 94, 1621–1634. 10.1016/j.biochi.2012.03.021 22503704

[B5] Brighigna L. Milocani E. Papini A. Vesprini J. L. (2006). Programmed cell death in the nucellus of Tillandsia (Bromeliaceae). Caryologia 59, 334–339. 10.1080/00087114.2006.1079793520978809

[B6] Bundrett M. C. Kendrick B. Peterson C. A. (1991). Efficient lipid staining in plant material with fluorol yellow 088 in polyethylene glycol-glycerol. Biotech. Histochem. 66, 111–116. 10.3109/10520299109110562 1716161

[B7] Cleary M. R. Holmes T. (2011). Formation of traumatic resin ducts in the phloem of western redcedar (*Thuja plicata*) roots following abiotic injury and pathogenic invasion by Armillaria ostoyae. IAWA J. 32, 351–359. 10.1163/22941932-90000063

[B8] Danti R. Barberini S. Pecchioli A. Di Lonardo V. Della Rocca G. (2014). The epidemic spread of *Seiridium cardinale* on Leyland cypress severely limits its use in the Mediterranean. Plant Dis. 98, 1081–1087. 10.1094/PDIS-12-13-1237-RE 30708785

[B9] Danti R. Della Rocca G. (2017). Epidemiological history of cypress canker disease in source and invasion sites. Forests 8, 1–25. 10.3390/f8040121

[B10] Danti R. Della Rocca G. Di Lonardo V. Pecchioli A. Raddi P. (2011). “Genetic improvement program of cypress: Results and outlook,” in Status of the Experimental Network of Mediterranean Forest Genetic Resources, eds. Besacier, C., Ducci, F., Malagnoux, M., and Souvannavong, O. (Silva Mediterranea, Rome: CRA SEL, Arezzo and FAO), 88–96.

[B11] Danti R. Della Rocca G. Panconesi A. (2013a). “Cypress canker,” in Infectious Forest Diseases, eds. P. Gonthier and G. Nicolotti (Wallingford, CT; Oxfordshire; Boston, MA: CABI), 359–75. 10.1079/9781780640402.0359

[B12] Danti R. Di Lonardo V. Pecchioli A. Della Rocca G. (2013b). ‘Le Crete 1' and ‘Le Crete 2': two newly patented *Seiridium cardinale* canker-resistant cultivars of *Cupressus sempervirens*. For. Pathol. 43, 204–210. 10.1111/efp.12016

[B13] Danti R. Raddi P. Panconesi A. Di Lonardo V. Della Rocca G. (2006). “Italico” and “Mediterraneo”: two *Seiridium cardinale* canker resistant cypress cultivars of *Cupressus sempervirens*. Hortsci. 41, 1357–1359. 10.21273/HORTSCI.41.5.1357

[B14] Danti R. Rotordam M. G. Emiliani G. Giovannelli A. Papini A. Tani C.. (2018). Different clonal responses to cypress canker disease based on transcription of suberin-related genes and bark carbohydrates' content. Trees. 32, 1707–1722. 10.1007/s00468-018-1745-5

[B15] David R. Carde J. P. (1964). Coloration differentielle des inclusions lipidiques et terpeniques des pseudophylles du Pin marictime on moyen du reactif Nadi. Comptes Rendus de l'Academie des Sciences Paris 258, 1338–1340.

[B16] Della Rocca G. Danti R. Raddi P. (2007). Le specie di cipresso nel mondo. Firenze: CNR-IPP.

[B17] Della Rocca G. Danti R. Williams N. Eyre C. Garbelotto M. (2019). Molecular analyses indicate that both native and exotic pathogen populations serve as sources of novel outbreaks of Cypress Canker Disease. Biol. Invasions. 21, 1–14. 10.1007/s10530-019-02022-9

[B18] Della Rocca G. Eyre C. A. Danti R. Garbelotto M. (2011). Sequence and simple-sequence repeat analyses of epidemic for the Mediterranean region. Phytopathol. 101, 1408–1417. 10.1094/PHYTO-05-11-0144 21879790

[B19] Della Rocca G. Osmundson T. Danti R. Doulis A. Pecchioli A. Donnarumma F.. (2013). AFLP analyses of California and Mediterranean populations of *Seiridium cardinale* provide insights on its origin, biology and spread pathways. For. Path. 43, 211–221. 10.1111/efp.12019

[B20] Della Rocca G. Posarelli I. Morandi F. Tani C. Barberini S. Danti R.. (2021). Different polyphenolic parenchyma cell and phloem axial resin duct-like structures formation rates in *Cupressus sempervirens* clones infected with *Seiridium cardinale*. Plant Dis. 105, 2801–2808. 10.1094/PDIS-01-21-0098-RE 33904337

[B21] Donaldson L.. (2020). Autofluorescence in plants. Molecules 25, 2393. 10.3390/molecules25102393 32455605 PMC7288016

[B22] Donaldson L. Williams N. (2018). Imaging and spectroscopy of natural fluorophores in pine needles. Plants. 7, 10. 10.3390/plants7010010 PMC5874599 29393922

[B23] Fahn A.. (1990). Plant Anatomy. Oxford: Pergamon Press.

[B24] Farahmand H.. (2020). The genus *Cupressus* L. mythology to biotechnology with emphasis on mediterranean cypress (*Cupressus sempervirens* L.). Hortic. Rev. (Am. Soc. Hortic. Sci.) 47, 213–287. 10.1002/9781119625407.ch5

[B25] Franceschi V. R. Krokene P. Krekling T. Christiansen E. (2000). Phloem parenchyma cells are involved in local and distant defense responses to fungal inoculation or bark-beetle attack in Norway spruce (Pinaceae). Am. J. Bot. 87, 314–326. 10.2307/2656627 10718992

[B26] Fujii T. Osumi K. Kubono T. (2018). Resin canals in “hiwada,” bark of hinoki (Chamaecyparis obtusa) as roofing material. Bull. Forest. Prod. Res. Instit. 17, 305–316.

[B27] Giuliani C. Pieraccini G. Santilli C. Tani C. Bottoni M. Schiff S.. (2020). Anatomical investigation and GC-MS analysis of “Coco de Mer”, Lodoicea maldivica (JF Gmel.) Pers. (Arecaceae). Chem. Biodivers. 17, e2000707. 10.1002/cbdv.202000707 33025751

[B28] Graniti A.. (1998). Cypress canker: a pandemic in progress. Annu. Rev. Phytopathol. 36, 91–114. 10.1146/annurev.phyto.36.1.91 15012494

[B29] Grundhöfer P. Niemetz R. Schilling G. Gross G. G. (2001). Biosynthesis and subcellular distribution of hydrolyzable tannins. Phytochemistry 57, 915–927. 10.1016/S0031-9422(01)00099-1 11423141

[B30] Hudgins J. W. Franceschi V. R. (2004). Methyl jasmonate-induced ethylene production is responsible for conifer phloem defense responses and reprogramming of stem cambial zone for traumatic resin duct formation. Plant Physiol. 135, 2134–2149. 10.1104/pp.103.037929 15299142 PMC520785

[B31] Krekling T. Franceschi V. R. Berryman A. A. Christiansen E. (2000). The structure and development of polyphenolic parenchyma cells in Norway spruce (*Picea abies*) bark. Flora 195, 354–369 10.1016/S0367-2530(17)30994-5

[B32] Krekling T. Franceschi V. R. Krokene P. Solheim H. (2004). Differential anatomical response of Norway spruce stem tissues to sterile and fungus infected inoculations. Trees (Berl.) 18, 1–9. 10.1007/s00468-003-0266-y

[B33] Krokene P. Nagy N. E. Krekling T. (2008a). “Traumatic resin ducts and polyphenolic parenchyma cells in conifers”, in Induced Plant Resistance to Herbivory, ed. A. Schaller (Dordrecht: Springer), 147–169.

[B34] Krokene P. Nagy N. E. Solheim H. (2008b). Methyl jasmonate and oxalic acid treatment of Norway spruce: anatomically based defense responses and increased resistance against fungal infection. Tree Physiol. 28, 29–35. 10.1093/treephys/28.1.29 17938111

[B35] Kusumoto D. Suzuki K. (2003). Spatial distribution and time course of polyphenol accumulation as a defense response induced by wounding in the phloem of Chamaecyparis obtusa. New Phytol. 159, 167–173. 10.1046/j.1469-8137.2003.00775.x 33873686

[B36] Li N. Dong Y. Lv M. Qian L. Sun X. Liu L.. (2021). Combined analysis of volatile terpenoid metabolism and transcriptome reveals transcription factors related to terpene synthase in two cultivars of Dendrobium officinale flowers. Front. Genet. 12, 661296. 10.3389/fgene.2021.661296 33968137 PMC8101708

[B37] Lison L.. (1960). Histochimie et cytochimie animales, Vol I. Paris: Gauthier-Villars.

[B38] Luchi N. Ma R. Capretti P. Bonello P. (2005). Systemic induction of traumatic resin ducts and resin flow in Austrian pine by wounding and inoculation with Sphaeropsis sapinea and Diplodia scrobiculata. Planta 221, 75–84. 10.1007/s00425-004-1414-3 15843966

[B39] Madar Z. Gottlieb H. E. Cojocaru M. Riov J. Solel Z. Sztejnberg A. (1995). Antifungal terpenoids produced by cypress after infection by *Diplodia pinea* f. sp. cupressi. Phytochemistry 38, 351–354. 10.1016/0031-9422(94)00575-E

[B40] Madar Z. Liphschitz N. (1989). Historical studies of Cupressus sempervirens L. affected by *Diplodia pinea* f.sp. *cupressi* and Seiridium cardinale. IAWA J. 10, 183–192.

[B41] Mehrshahi P. Stefano G. Andaloro J. M. Brandizzi F. Froehlich J. E. DellaPenna D. (2013). Transorganellar complementation redefines the biochemical continuity of endoplasmic reticulum and chloroplasts. Proc. Nat. Ac. Sci. 110, 12126–12131. 10.1073/pnas.1306331110 23818635 PMC3718160

[B42] Moriondo F.. (1972). Cancro del cipresso da Coryneum cardinale Wag. I. La progressione del processo infettivo nei tessuti caulinari. Accad. Ital. Sci. Forest. XXI, 399–426.

[B43] Mosti S. Ross Friedman C. Pacini E. Brighigna L. Papini A. (2013). Nectary ultrastructure and secretory modes in three species of *Tillandsia* L. (Bromeliaceae) that have different pollinators. Botany. 91, 786–798. 10.1139/cjb-2013-0126

[B44] Mutto S. Panconesi A. (1987). Ultrastructural modifications in *Cupressus sempervirens* tissues invaded by *Seiridium cardinale*. Eur. J. For. Pathol. 17, 193–204. 10.1111/j.1439-0329.1987.tb01016.x

[B45] Nagy N. E. Franceschi V. R. Solheim H. Krekling T. Christiansen E. (2000). Wound-induced traumatic resin duct development in stems of Norway spruce (Pinaceae): anatomy and cytochemical traits. Am. J. Bot. 87, 302–313. 10.2307/2656626 10718991

[B46] Panconesi A. Raddi P. (1990). Agrimed n. 1 e Bolgheri: due nuove selezioni resistenti al cancro. Cellulosa e carta. 42, 47–52.

[B47] Papini A. Mosti S. Van Doorn W. G. (2014). Classical macroautophagy in *Lobivia rauschii* (Cactaceae) and possible plastidial autophagy in *Tillandsia albida* (Bromeliaceae) tapetum cells. Protoplasma 251, 719–725. 10.1007/s00709-013-0567-y 24158376

[B48] Phillips M. A. Croteau R. B. (1999). Resin-based defenses in conifers. Trends Plant Sci. 4, 184–190. 10.1016/S1360-1385(99)01401-6 10322558

[B49] Ponchet J. Andreoli C. (1990). “Compartmentalization and reaction in the host”, in: *Progress in EEC Research on Cypress Diseases. Results of the Agrimed Project (1980–88)*, ed J. Ponchet (Luxembourg: Commission of the European Communities EUR 12493), 96–111.

[B50] Raddi P. Panconesi A. (1981). Cypress canker disease in Italy: biology, control possibilities and genetic improvement for resistance. Eur. J. Forest Pathol. 11, 340–347. 10.1111/j.1439-0329.1981.tb00104.x

[B51] Ribeiro V. C. Leitão C. A. E. (2020). Utilisation of Toluidine blue O pH 4.0 and histochemical inferences in plant sections obtained by free-hand. Protoplasma 257, 993–1008. 10.1007/s00709-019-01473-0 31865451

[B52] Spanos K. A. Pirrie A. Woodward S. Xenopoulos S. (1999). Responses in the bark of *Cupressus sempervirens* clones artificially inoculated with *Seiridium cardinale* under field conditions. Eur. J. For. Pathol. 29, 135–142. 10.1046/j.1439-0329.1999.00136.x

[B53] Spurr A. R.. (1969). A low-viscosity epoxy resin embedding medium for electron microscopy. J. Ultrastruct. Res. 26, 31–43. 10.1016/S0022-5320(69)90033-1 4887011

[B54] Suto Y.. (1998). Traumatic resin-canal formation caused by inoculation with Cistella japonica in secondary phloem of Chamaecyparis obtusa. J. For. Res. 3, 99–102. 10.1007/BF02760309

[B55] Trapp S. Croteau R. (2001). Defensive resin biosynthesis in conifers. Annu. Rev. Plant Biol. 52, 689–724. 10.1146/annurev.arplant.52.1.689 11337413

[B56] Van Wijk K. J. Kessler F. (2017). Plastoglobuli: plastid microcompartments with integrated functions in metabolism, plastid developmental transitions, and environmental adaptation. Annu. Rev. Plant Biol. 68, 253–289. 10.1146/annurev-arplant-043015-111737 28125283

[B57] Wagener W. W.. (1928). Coryneum canker of Cypress. Science 67, 584. 10.1126/science.67.1745.584.a 17750085

[B58] Wagener W. W.. (1939). The canker of Cupressus induced by *Corineum cardinale*. sp. J. Agric. Res. 58, 1–46.

[B59] Xenopoulos S. Andréoli C. Panconesi A. Pinto Ganhao J. Tuset J. (1990). “Importance of cypress,” in: *Progress in EEC Research on Cypress Diseases. Results of the Agrimed Project (1980–88)*, ed J. Ponchet (Luxembourg: Commission of the European Communities EUR 12493),1–13.

[B60] Yamanaka K.. (1989). Formation of traumatic phloem resin canals in Chamaecyparis obtuse. IAWA J. 10, 384–394.

[B61] Yao N. Eisfelder B. J. Marvin J. Greenberg J. T. (2004). The mitochondrion - an organelle commonly involved in programmed cell death in *Arabidopsis thaliana*. Plant J. 40, 596–610. 10.1111/j.1365-313X.2004.02239.x 15500474

[B62] Zulak K. G. Bohlmann J. (2010). Terpenoid biosynthesis and specialized vascular cells of conifer defense. J. Integr. Plant Biol. 52, 86–97. 10.1111/j.1744-7909.2010.00910.x 20074143

